# Misdiagnosis of SARS-CoV-2: A Critical Review of the Influence of Sampling and Clinical Detection Methods

**DOI:** 10.3390/medsci9020036

**Published:** 2021-05-25

**Authors:** Daniel Keaney, Shane Whelan, Karen Finn, Brigid Lucey

**Affiliations:** 1 Department of Biological Sciences, Munster Technological University, Bishopstown, T12 P928 Cork, Ireland; daniel.keaney@mycit.ie (D.K.); Swhelan1@mycit.ie (S.W.); brigid.lucey@cit.ie (B.L.); 2 Department of Biopharmaceutical and Medical Science, Galway-Mayo Institute of Technology, Old Dublin Road, H91 DCH9 Galway, Ireland

**Keywords:** COVID-19, SARS-CoV-2, Rt-PCR, sampling, detection methods, antibody tests, antigen tests, symptomatic, asymptomatic, diagnosis

## Abstract

SARS-CoV-2 infection has generated the biggest pandemic since the influenza outbreak of 1918–1919. One clear difference between these pandemics has been the ability to test for the presence of the virus or for evidence of infection. This review examined the performance characteristics of sample types *via* PCR detection of the virus, of antibody testing, of rapid viral antigen detection kits and computerised tomography (CT) scanning. It was found that combined detection approaches, such as the incorporation of CT scans, may reduce the levels of false negatives obtained by PCR detection in both symptomatic and asymptomatic patients, while sputum and oral throat washing sample types should take precedence over swabbing when available. Rt-PCR assays for detection of the virus remain the gold-standard method for SARS-CoV-2 diagnosis and can be used effectively on pooled samples for widespread screening. The novel Oxford antibody assay was found to have the highest sensitivity and specificity of four currently available commercial antibody kits but should only be used during a specific timeframe post-symptom onset. Further research into transmission modes between symptomatic and asymptomatic patients is needed. Analysis of the performance characteristics of different sampling and detection methods for SARS-CoV-2 showed that timing of sampling and testing methods used can greatly influence the rate of false-positive and false-negative test results, thereby influencing viral spread.

## 1. Introduction

Real-time polymerase chain reaction (Rt-PCR)-based testing is currently the most common form of sample testing for the presence of SARS-CoV-2 for both symptomatic and asymptomatic patients and is seen as a reliable method for the detection of virus nucleic acid [1]. A 2015 meta-analysis carried out in Shanghai to evaluate the effectiveness of Rt-PCR for diagnosing novel coronavirus infections, based on past pandemics, concluded that PCR is an effective tool in disease diagnosis [1].

A Canadian study by LeBlanc et al. (2020) in a comparison of 23 PCR laboratory-developed tests (LDTs) used for the detection of SARS-CoV-2 and some commercially available real-time reverse transcriptase PCR (RT-PCR) assays has found that, overall, there was little variation in the limit of detection between the LDTs (which tended to be between 3.4 and 4.5 log10 copies/mL) and the commercial assays and that few of the assays showed a reduced sensitivity [2].

Unfortunately, the use of cycle threshold values for positive qualitative Rt-PCR tests for denoting viral load in the patient is not helpful as this method depends on the sensitivity of the assay being performed and does not give an absolute value for the viral copy number in the sample. There is a scarcity of reports of the expected viral load in respiratory samples of patients with COVID-19 disease during the course of the infection. One study published by Pan et al. (2020) in the Lancet, examining (only) two patients, has shown when using a PCR assay that amplified the N-gene in a quantitative RT-PCR that viral loads in throat swab and sputum samples peaked at approximately 5–6 days after symptom onset and contained approximately 10^4^ to 10^7^ copies per mL during that period.

Notwithstanding the scarcity of quantitative assays, another approach towards assessing the accuracy of testing methods for COVID-19 was a systematic review of the accuracy of COVID-19 diagnostic tests consisting of 34 case studies and 12,057 COVID-19-confirmed cases, reported false negatives between 2% and 29% (equating to a sensitivity of 71–98%) [3], thus signalling the need for a comprehensive analysis focusing on the reasons contributing to misdiagnosis.

## 2. Symptomatic and Asymptomatic Patients

Asymptomatic patients are those who are actively carrying the live replicating virus, yet not presenting with any of the typical characteristic symptoms, in contrast to symptomatic patients who do. Due to the lack of physical indicators which may alert a carrier to the presence of the virus within their system, these asymptomatic patients are a cause for concern with regard to the continual, unknown transmission of the virus. In some instances, methods used to detect the presence of the virus may even be less sensitive for asymptomatic patients than those who are symptomatic [4,5].

As shown in Table 1, it is evident that across both single-case studies and meta-analyses, a sizeable proportion of all cases are asymptomatic. In the study carried out by Sayampanathan (2021), 3790 close contacts were also observed for the development of COVID-19. Overall, 89 people tested positive. Of those, 50 people (56%) were asymptomatic, while 39 people (44%) were symptomatic. In general, this study concluded that symptomatic cases were 3.85-fold more likely to transmit the virus in comparison to asymptomatic cases [6]. It must be noted that there is a lack of published literature surrounding comparative transmission models in asymptomatic and symptomatic patients, but it has been deduced that viral shedding of patients with laboratory-confirmed COVID-19 has shown to peak, on or before symptom onset, and a substantial proportion of transmission likely occurs before the first symptoms are present [7]. It can be recommended that robust comparative experimentation should be conducted on the differences in transmission of both asymptomatic and symptomatic patients.

The development of symptoms in asymptomatic carriers was found to be a common occurrence in most studies. The occurrence of symptoms occurred in the following studies as shown: 21/110 (19.1%) [8]; 88/180 (48.9%) [7] across 10 studies and in 36% of asymptomatic obstetric patients [11].

Interestingly, across 11 case studies focused on 1152 COVID-19-positive children, the amount of asymptomatic cases was found to be 27.7%, which was noted to be much higher than patients from all other age groups [7]. Worryingly, there appears to be an informed mindset that children are less susceptible to acquiring and transmitting COVID-19 infection due to numerous physiological and socio-economic reasons [12,13,14], yet evidence suggests that children are the most likely age group to be asymptomatic carriers. Therefore, it can be recommended that all scientific outreach surrounding children and COVID-19 should not perpetuate an idea of age-based ‘safety’ from infection and transmission of the virus.

## 3. Examination of Sampling Techniques for Large Populations

When it comes to monitoring and containing the spread of an infectious disease, it could be proposed that it is more efficient to identify those who are not infected as opposed to determining those that are, followed by contact tracing and trying to stop the spread of the disease any further. As such, it is important to consider all the available methods and protocols involved in the testing of large populations, and find ways to implement them.

### 3.1. Molecular Tests: PCR and LAMP Testing

While PCR testing can be considered the gold-standard test for detecting the presence of SARS-CoV-2 nucleic acid within a sample, it is necessary to assess the assay design. Due to the nature of PCR testing, the target sequence being amplified is one of most important design details that needs to be addressed in order to ensure accurate diagnosis when facing new variants and mutations of the virus. Shortly after the emergence of COVID-19 in China, in 2020, the World Health Organisation (WHO) released a set of diagnostic Rt-PCR-based diagnostic schemes for COVID-19 based on the amplification of a number of different viral genes—notably, the RNA-dependent RNA polymerase-encoding (*RdRp*) gene (the target with the most specificity), the viral nucleocapsid *N* gene and the envelope *E* gene (the target with the highest sensitivity) [15,16]. To determine that the WHO-released diagnostic scheme target regions were not open to high levels of mutation, several *in silico* studies to evaluate the potential for mutation to occur have been carried out. Overall, a mismatch was detected in the Charité-ORF1b primer, while a high frequency of mutation was determined in the forward *N* gene primer released by the China CDC [17,18,19,20,21,22]. Subsequently, further independent work led to the withdrawal of publicly available sets of primers with the exception of forward N China CDC primer and set N3 of the US CDC [15]. Since then, Peñarrubia (2020) analysed nine publicly available primers/probes, with more than 30,000 genomes. A high frequency of mutation was found within the regions of interest and a conclusion was drawn based on adopting multiple targeting approaches which may help bypass the risk in losing assay sensitivity [17]. A further bioinformatics approach to determine the presence of mismatches between PCR assay primers and probes among selected COVID-19 genomes included the more recent UK B.1.1.7 and the South African 501Y.V2 variants. The UK variant has a large number of mutations in its spike protein, although assays targeting the S-gene associated with the spike protein are not commonplace. The UK variant lineage showed a higher rate of molecular evolution, compared to other COVID-19 lineages, outside of the S gene. When subjected to a basic local alignment search tool (BLAST) test, a perfect match was found between B.1.1.7 and the probes released by the WHO. A similar analysis was carried out on the South African variant and only two mismatches were found involving the central parts of China CDCN forward primer and the Japan National Institute of Infectious Diseases N reverse primer [15]. Given the work conducted both physically and *in silico*, it appears that the variability occurring within the COVID-19 population may have a minimal or no effect on the sensitivity of existing detection systems based on molecular markers, thus reaffirming Rt-PCR as the gold-standard test, even in the face of particularly publicised variants such as B.1.1.7 and 501Y.V2.

When observing molecular tests, it is important to highlight the emergence of a newer type of test—LAMP testing. Reverse transcriptase-loop-mediated isothermal amplification (RT-LAMP) is a single-tube technique for the amplification of RNA, while LAMP testing alone can be used to detect DNA [23]. It is a low-cost alternative in the detection of disease. In contrast to PCR technology, which amplifies in the presence of alternating temperature cycling steps, isothermal amplification is carried out at a constant temperature and does not require specialist equipment such as a thermal cycler. This is a significant benefit for low-income or middle-income countries when it comes to rapid detection of the virus. LAMP testing has also been observed to be more resistant to PCR inhibitors in complex samples, such as blood [24,25,26,27]. Portable and user-friendly RT-LAMP kits have been successfully used in the detection of COVID-19, with available results in 30 min [28]. The system was tested using 10 clinical samples, and was able to detect SARS-CoV-2 from these clinical samples by distinguishing positive samples from negative samples after 30 min. More importantly, these tests were confirmed by RT-PCR [28].

Another study examining the efficacy of variplex™ RT-LAMP has also shown promising results. The kit showed a sensitivity of 75% compared to LightMix E gene RT-PCR but contrary to the latter it produced no false-positive results. For the evaluation of samples from respiratory secretions analysis depicted a moderate agreement between the variplex™ RT-LAMP conducted on unprocessed samples and Allplex™ and VIASURE RT-PCRs (Cohen’s κ ranging from 0.52–0.56). Using the approach to define a sample as true-positive when at least two assays gave a positive result the reported clinical sensitivities were as follows: 76.3% for variplex™, 84.2% for Allplex™ and 68.4% for VIASURE. However, when results of RT-PCR and RT-LAMP were combined diagnostic sensitivity was increased to 92–100% [29] making RT-LAMP employment, especially in low-income regions of the world, a particularly useful method of virus detection.

While discussing the molecular testing of COVID-19, it is relevant to explore the correct method of sampling, which may be the difference between a successful or missed diagnosis. A systematic review into the accuracy of COVID-19 tests, consisting of 34 case studies and 12,057 COVID-19-confirmed cases, reported false negatives between 2% and 29% (equating to a sensitivity of 71–98%) [3]. Swabbing is a commonplace practice in the collection of samples for molecular testing. Sample collection swabs must be synthetic in nature (polyester, rayon, or dacron), as directed by the centre of disease control (CDC). Where sampling involves the use of swabs, cotton or calcium alginate tipped, or wooden shaft swabs are not acceptable and can cause interference with PCR reactions [30]. Interestingly, swab manufacturer plays an equally important role in obtaining accurate results. Research analysing the volume of viral material absorbed and released from differently manufactured nylon flocked swabs, showed a statistically significant difference in viral absorption and elution levels [31]. Similarly, ultra-absorptive nanofiber swabs have shown to help reduce the likelihood of false negatives and identifies SARS-CoV-2 at a 10× lower viral concentration compared to flocked and cotton swabs [32].

It must be noted, that while the physio-chemical properties of PCR interference, based on swab material, have been examined, the sensitivity, specificity, positive and negative predictive values in the detection of COVID-19, based on swab material, has not yet been scientifically published. This is an important factor to consider for future studies when examining samples intended for PCR analysis.

### 3.2. Sample Pooling Using Rt-PCR

Methods of sample pooling, both prior to and following RNA extraction for Rt-PCR detection have been investigated as a means to reduce the number of tests carried out by laboratories and thereby conserve test kits, resources and labour time. If testing the pool results in a negative result, then in turn all the sample specimens which contributed the pool can be presumed negative to the limit of detection defined by the verification stage conducted when preparing to use the testing method. If testing the pool returns a positive result, then all the contributing specimens can be retested individually. The availability of testing reagents is a limiting factor, however, and the economic difficulty of implementing widespread testing of asymptomatic populations is a reality, despite the utility this would bring to pandemic management when prevalence of infection is low.

When gathered in pools, the ratio of positive samples to negative is unknown prior to testing, so determining the effect of dilution on the cycle threshold (Ct) number is necessary. The resource saving of high sample pools must be balanced with the loss of sensitivity seen as pool sizes and dilution of positive samples increase. The Ct number at which fluorescence is detected must remain under 40 cycles for a positive result according to the WHO. Positive samples must be detected as positive in under 40 cycles for a pooling strategy to be valid [33].

Nasopharyngeal swabs can be collected into viral transport medium (VTM) and pooled prior to RNA extraction to create a pre-lysis pool, reducing the test kits required for the extraction step, which is a reagent-costly bottleneck. The loss in sensitivity in 10 sample pools made up of nasopharyngeal swab samples in VTM compared to 5 sample pools appears to be significant, with 5 sample pooling maintaining 95.5% concordance with individual testing on samples with Ct values of ≤33 while 10 sample pools dropped to 66%, potentially leading to unacceptable levels of false negatives when targeting the *E* gene [34]. False negatives were elsewhere reported when targeting the ORF1ab and *N* gene in 6 and 10 sample pools constructed from VTM, although the mean Ct values were 36.68 and 37.12. This was speculated to result from low viral load of individual samples comprising the pool [35].

The pooling of oropharyngeal swabs at the point of collection prior to RNA extraction is a promising strategy. When pools of 6 and 10 swabs in VTM were compared, the mean Ct values of the ORF1ab and *N* gene were 37.73 and 37.08 in both pools and were detected in all cases without false-negatives [35]. However, the drawback to this method is that two swabs will need to be taken from each patient to allow for retesting. Saliva samples have been pooled prior to RNA extraction also and sensitivity was found to decrease as pool size increased ranging from a 2.2 Ct increase for 5 sample pools to a 3.6 Ct increase for 20 sample pools, but was not significantly different from the loss in sensitivity seen when RNA extracts were pooled [36].

RNA can also first be extracted from VTM and then extracts can be pooled. Pooling RNA extractions in pools of 2, 4, 6 and 8 maintained 100% concordance with individual testing when targeting the *E* gene [37]. The Ct value did increase above individual testing but remained ≤40 cycles. Following a similar pooling protocol, a Spanish study reported that Ct values tested by Rt-PCR were delayed by 5 cycles for 20 sample pools and 2.85 cycles for 5 sample pools. Sensitivity for the *E*, *RdRP* and *N* gene was 88.5%, 84.6% and 96.1% in 20 sample pools, whereas 5 sample pools had 92.9% sensitivity for all 3 targets [38].

It is important to determine the effective limits of detection when pooling samples for detection using Rt-PCR. Using a plasmid control containing the *N* gene of SARS-CoV-2 and diluted to concentrations containing 100,000, 10,000, 1000 and 100 copies, it was found that the limits of detection were 100–1000 copies for the N1 site and 10–100 copies for the N2 site [39]. Spike-in assays using samples from SARS-CoV-2 patients with varying viral loads pooled in ratios of 1:4, 1:9 and 1:19 have shown that the N2 site is more detectable than the N1 site when subjected to Rt-PCR, where the N2 site was detectable even in samples with a low viral load (3.6 log10) in all three pools, while the N1 site was detectable only when samples of high or moderate viral load were used [39].

The *N* and *RdRp* genes have also been successfully detected using Rt-PCR in 8 sample pools prepared prior to RNA extraction. The Ct values have been reported between 29.0 and 31.8, with expected increases when compared to individually tested samples due to the dilution. The same study also investigated the detectability of extracted RNA samples in pools with sizes up to 64 samples, finding a single positive sample was detectable in pools up to 32 samples with a false-negative rate of 10% [40].

### 3.3. An Alternative Method for Sampling Large Populations: Estimating the Value of Sample Self-Collection

One way of ensuring that the spread of the disease is controlled is by allowing the self-collection of diagnostic samples by an entire population. A study into the willingness of patients to self-collect samples, along with their ability to collect quality samples was carried out by Valenine-Graves and Sullivan in June 2020. In the study, 153 USA adults were instructed on how to collect, package and post-three diagnostic samples; saliva, oropharyngeal swabs and dried blood spot cards while being observed by a clinician through a telehealth session. The majority of the participants (>84%) reported that collecting, packing and shipping of saliva, oropharyngeal swabs, and dried blood spot samples were acceptable. Of the participants examined, 87% reported being ‘confident’ or ‘very confident’ that the samples they collected were sufficient for laboratory analysis. There were no differences in acceptability for any specimen type, packing and shipping, or confidence in samples by gender, age, race/ethnicity, or educational level, thus showing a significant competency within the general public to carry out a self-sampling regime, which would allow for a national breakdown of infections and the spread of disease [41].

### 3.4. Other Diagnostic Tests Used

#### 3.4.1. Antibody Tests

In response to infection, IgM first becomes detectable in the serum 2–3 days post-infection, followed by IgG; specifically 6 days in critical cases [42]. IgM can be used to locate early-stage infections while IgG could be used to infer post-infection immunity. The use of antibody-antigen specific enzyme-linked immunosorbent assay (ELISA) blood tests could provide significant improvements in the detection of COVID-19 prior to the development of symptoms and can be as efficient as a finger prick of blood [43,44,45,46,47]. However, it must be noted that the test results may be impacted by the following; the patients may be seropositive yet negative by Rt-PCR, results reflect clearance of an earlier milder infection, a subgroup of the patients with a positive result from molecular assays for COVID-19 infection may be seronegative due to a lag in antibody production following infection, patients may be immunosuppressed, and there may be limitations in the sensitivity or specificity of the assays themselves [48]. There is also a chance that other coronavirus antibodies may be detected within the body and lead to a false positive.

Due to the constantly changing knowledge surrounding SARS-CoV-2, newer information is constant. Preliminary studies carried out by Tan et al. (2020) [49] and Zhao et al., (2020) [50] determined that IgM was detected on day 7 and peaked on day 28 (across 28 patients), and IgG appeared by day 10 and peaked on day 49 (45 patients); that seroconversion among 173 patients took place at median times of 12 (IgM), 14 (IgG), and 11 (neutralizing antibodies) days. Meanwhile, a systematic review conducted by Whelan et al. (2020) determined that IgM peaked at 15, 15 and 24 days post-symptom onset in mild, critical and severe patients, respectively, while IgG peaked at 30, 24 and 20 days post-symptoms onset in mild, critical and severe cases, respectively.

Coronavirus is comprised of four structural proteins: the spike protein (S), which is immunodominant and consists of two subunits, the nucleocapsid (N), whose protein is smaller than the S unit, is also immunogenic, the envelope protein (E) and the membrane protein (M). The N protein induces antibody responses sooner than S proteins and make for an attractive diagnostic marker when designing antibody assays [51,52,53,54,55,56,57,58,59,60,61]. Therefore, antibody tests based on antibodies to N proteins may be more useful than those detecting S proteins as they cover a bigger timespan.

**Figure 1:**
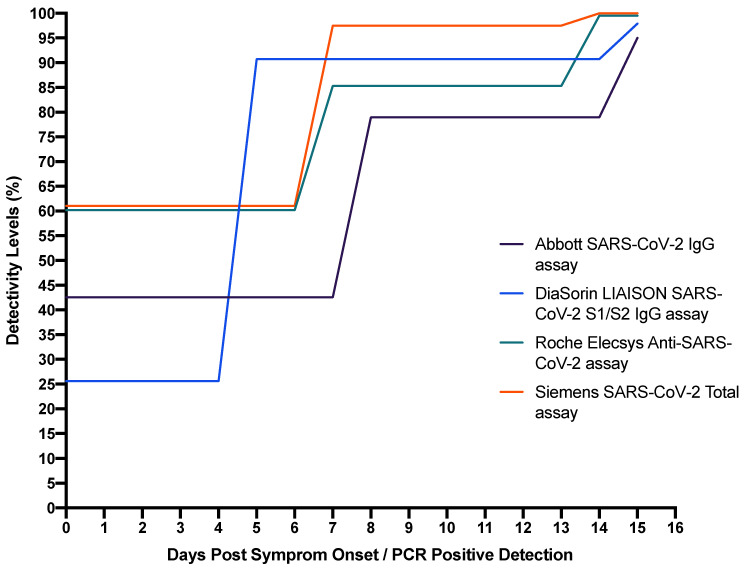
The time sensitive changes of antibody detectivity levels within COVID-19-positive patients over time when tested using available and marketed commercial kits—Abbott, DiaSorin, Roche and Siemens.

An investigation into the performance of four high-throughput commercial SARS-CoV-2 antibody immunoassays; SARS-CoV-2 IgG assay (Abbott, Chicago, IL, USA), LIAISON SARS-CoV-2 S1/S2 IgG assay (DiaSorin, Saluggia, Italy), Elecsys Anti-SARS-CoV-2 assay (Roche, Basel, Switzerland), SARS-CoV-2 Total assay (Siemens, Munich, Germany), and a novel 384-well ELISA (the Oxford immunoassay) was performed by Ainsworth (2020). The targets of the assays are as follows; the Abbott SARS-CoV-2 IgG assay binds to the receptor binding domain of the S1 subunit of the spike protein; the DiaSorin LIAISON SARS-CoV-2 S1/S2 IgG assay binds to the IgG antibodies anti-Trimeric Spike glycoprotein of SARS-CoV-2; the Roche Elecsys Anti-SARS-CoV-2 assay detects the N protein antigen; the Siemens SARS-CoV-2 Total assay detects IgG and IgM antibodies by immunofluorescence; while the Novel Oxford immunoassay is an indirect ELISA, measuring serum IgG against trimeric spike protein. As seen in Table 2, it was found that the manufacturers’ threshold for the Abbott assay sensitivity was 92.7% (95% CI 90.2–94.8) and specificity was 99.9% (99.4–100%); for the DiaSorin assay, sensitivity was 96.2% (94.2–97.7) and specificity was 98.9% (98.0–99.4); for the Oxford immunoassay, sensitivity was 99.1% (97.8–99.7) and specificity was 99.0% (98.1–99.5); for the Roche assay, sensitivity was 97.2% (95.4–98.4) and specificity was 99.8% (99.3–100); and for the Siemens assay, sensitivity was 98.1% (96.6–99.1) and specificity was 99.9% (99.4–100%) [62]. While the Siemens and Oxford assays achieved a sensitivity of at least 98% without further optimisation, it was noted in the study that the high sensitivities and specificities for the other assays could only be achieved by threshold adjustment or testing samples that were taken 30 days post-symptom onset. This is not something that is routinely performed in most laboratories undertaking serological testing, especially during times with increased numbers of samples. It is also noted, as is evident in Figure 1 (which excludes non-commercial kits), that the percentage detection of these kits changes over time as antibodies are created. Therefore, it is recommended that these commercial kits must only be used at either time-specific points, such as 14 days post-symptoms onset onwards, to help detect the highest level of antibodies, or only as a complimentary diagnostic method in addition to more suited tests, such as Rt-PCR. Given the data in Table 2, it could be also recommended that the novel Oxford test be used as the gold-standard antibody test due to having the highest overall sensitivity and specificity.

#### 3.4.2. Antigen Tests and Their Use for Lateral Flow Testing

Rapid antigen tests may be employed to detect the presence of viral antigens to COVID-19 [63,64]. These have been employed in the past in many formats such as in the form of colorimetric enzyme immunoassays, in 2004 for the detection of SARS-CoV, and an enhanced chemiluminescent immunoassay for SARS-CoV in 2005 [63,65]. In particular, the *Abbott BinaxNOW™* COVID-19 rapid antigen test was tested at a public plaza site of ongoing community transmission in San Francisco.

Among 878 subjects tested, 3% (26/878) were positive by Rt-PCR, of which 15/26 had a Ct < 30, indicating high viral load. A total of 40% (6/15) of Ct < 30 were asymptomatic. Using this Ct < 30 threshold for Binax-CoV2 evaluation, the sensitivity of the Binax-CoV2 was 93.3% (14/15), 95% CI: 68.1–99.8%, and the specificity was 99.9% (855/856), 95% CI: 99.4–99.9% [66].

A more recently approved antigen test, by the Food and Drug Administration, is the *LumiraDx* SARS-CoV-2 antigen test. It is based on a microfluidic immunofluorescent assay design which aims to give results in as little as 11 min. Clinical testing has shown that the microfluidic test offers 97.6% sensitivity and 96.6% specificity [67]. When compared to Rt-PCR sampling it was found to have a positive agreement of 100% from days 0 to 3 post-symptom onset, 98.2% on day 4, 98.4% on day 5, 98.5% on day 6, 98.6% on day 7, 98.7% on days 8 to 10, 98.8% on day 11 and 97.6% on day 12. Out of the examined antigen tests, it is clear that the *LumiraDx* SARS-CoV-2 antigen test is currently the best on the market and could easily be employed in a large-scale testing regime, in place of the *Innova SARS-CoV-2 Antigen Rapid Qualitative Test*, as was performed with a lateral flow testing pilot in the UK.

Lateral flow tests are designed to distinguish/identify asymptomatic/symptomatic people from healthy individuals, and is planned to be implemented across the UK as a part of a mass-testing plan after a successful pilot in Liverpool. This test is created in a similar style to a pregnancy test (also known as a rapid diagnostic test) and the result can be obtained in 10–30 min. A liquid sample (nasopharyngeal, oropharyngeal, oral throat wash or sputum) is placed on an antigen test substrate, leading to a colorimetric result [48]. These tests are both relatively inexpensive and easy to use, hence the national roll-out announced in the UK in November 2020.

The *Innova SARS-CoV-2 Antigen Rapid Qualitative Test* carried out in Liverpool reviewed 8774 tests across multiple demographics. It found an overall sensitivity of 76.8%, but this rose to over 95% in individuals with high viral loads. The overall specificity of the test was reported as 99.68%, meaning a false positive rate of 0.32% (22/6967 tests) [68]. However, the Cochrane review (consisting of 22 publications reporting on a total of 18 study cohorts with 3198 unique samples, of which 1775 had confirmed SARS-CoV-2 infection) found overall that while the diagnostic accuracy of different branded rapid antigen tests differed, these tests generally had a higher sensitivity for symptomatic patients, in comparison to those who are asymptomatic [4,5], likely due to lower viral load in the latter population [42]. The Cochrane analysis deemed that the sensitivity of lateral flow tests in symptomatic cases, on average, was 72%, while the sensitivity for asymptomatic cases was, on average, 58% [5]. It must be noted the number of asymptomatic cases tested overall was 10-fold lower than symptomatic. Therefore, definite conclusions could not be drawn based on the figures [4].

The evaluation carried out in Liverpool found that the test performed best when used by laboratory scientists, where the sensitivity was 79% (156/197 positive: 79.2% (95% confidence interval 72.8% to 84.6%)).

Sensitivity dropped to 73% when used by trained health care staff (92/126 positive: 73.0% (64.3% to 80.5%)) and to 58% with self-trained members of the public (214/372 positive: 57.5% (52.3% to 62.6%)). With a sensitivity of 58% and specificity of 99.6%, if 100,000 people were tested, statistically the test would find 630 positives—of which only 230 would actually have COVID-19, while 400 would be false positive. With this degree of false positivity, lateral flow testing may be best suited to localised communities for fast detection and halting the spread of the disease [68], due to the lower levels of sensitivity and specificity.

While the onus is with the clinical labs to verify/validate the assays in order to warrant their employment, it is also noted how the manufacturers of these commercial kits do not state their limits of detection. This not only hinders the development of scientific research surrounding the virus, but may also influence the front-line in regard to the testing of patients who are asymptomatic, or that may not have high titres of the molecular bio-markers within their system due to variables such as the stage progression of the disease, or the individuals physiological characteristics. Therefore, it could be recommended that the limit of detection of these kits be made available in an independent, peer-reviewed publication that includes all the possible caveats surrounding the kits’ use.

#### 3.4.3. Computed Tomography Scanning—An Alternative to Laboratory-Based Testing

A computed tomography (CT) scan is a medical imaging procedure that uses computer-processed combinations of different X-ray measurements taken from different angles to create a clear and identifiable image to aid in diagnosis. It must be noted that there are limitations associated with the use of CT scans. Ground-glass artefacts that appear on CT scans are not fully indicative of COVID-19 and may be associated with other diseases (such as hydrostatic pulmonary edema, interstitial lung disease and connective tissue disease [69]), therefore total reliance on CT scans alone is not enough for accurate diagnosis. Nonetheless, CT scans have been included in many sets of data surrounding the testing for COVID-19 and will be analysed as such.

In Wuhan, 1014 patients had been confirmed to have the virus by Rt-PCR and were also examined using CT methods. Out of 1014 patients, 59% (601/1014) had positive Rt-PCR results, and 88% (888/1014) had positive chest CT scans. The sensitivity of chest CT suggests that COVID-19 was 97% accurate (95% CI, 95–98%, 580/601 patients) based on positive Rt-PCR results. In patients with negative Rt-PCR results, 75% (308/413) had positive chest CT findings; of 308, 48% were considered as highly likely cases, with 33% as probable cases [70].

Other investigations support the efficacy of CT scans. A study comprising 104 cases of COVID-19 were confirmed via Rt-PCR, the results were as follows; 76 (73%) were asymptomatic, 41 (54%) of which had lung opacities on their CT scans. The other 28 (27%) cases were symptomatic, 22 (79%) of which had abnormal CT findings. Symptomatic cases showed lung opacities and airway abnormalities on CT more frequently than asymptomatic cases [lung opacity; 22 (79%) in comparison to 41 (54%), airway abnormalities; 14 (50%) in comparison to 15 (20%)]. Asymptomatic cases showed more ground-glass opacity (an area of increased attenuation in the lung on CT scans) over consolidation (83%), while symptomatic cases more frequently showed consolidation over ground-glass opacity (41%). The CT severity score was higher in symptomatic cases than asymptomatic cases [71].

The use of artificial intelligence (AI) in reading CT scans to detect COVID-19 has also been investigated in order to improve the efficiency of diagnosis [72]. After the assessment of 1065 CT scans of COVID-19 patients, the AI algorithm had scored an 89.5% detection accuracy, while a human-lead external panel had scored a 79.3% detection accuracy. This indicates that this testing method has potential to be revolutionised digitally and aid in stopping the spreading of the disease through fast diagnosis [72].

Upon review, when assessing the use of CT scans in testing for COVID-19, studies conclude that there is a high level of subclinical changes associated with COVID-19, compared to mild changes observed in asymptomatic patients; thus providing assurance that CT scans are reliable and can be efficiently used in tandem with other sampling methods when diagnosing both asymptomatic patients, as well as those showing symptoms [71]. Data suggest that using CT scans in combination with other testing methods offers a highly efficient and reliable means of detecting COVID-19 in both symptomatic and asymptomatic patients when compared to other independent test methods with high levels of false negatives of missed positives. This is due to the ability to examine the lungs in real time as the infection develops. This method is also non-invasive, yet takes time to complete, but with the use of artificial intelligence may also become a high-throughput screening method in the future. As a result, this method of sampling would be most efficient in hospitalised patients.

## 4. Examination of Sample Types Efficacy Used for Diagnosing COVID-19 Disease

### 4.1. Nasopharyngeal Swabs and Oropharyngeal Swabs

**Figure 2:**
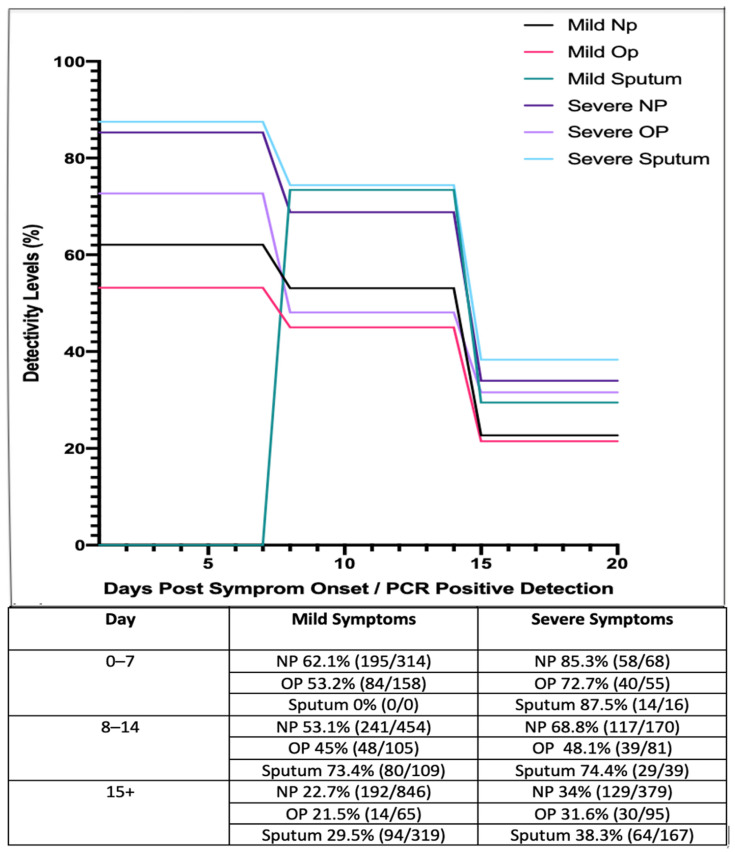
Longitudinal analysis of COVID-19 percentage positivity rates when sampled across 213 hospitals in China, over 15+ days, using nasopharyngeal (NP), oropharyngeal (OP) swabs and sputum samples (adapted from [73]).

A study from China examined 410 COVID-19 patients across 213 hospitals in China as they were subjected to intermittent NP, OP swabbing, and sputum sampling [73]. The results of these studies are presented in Figure 2 below.

These studies suggest that those with mild symptoms are less detectable as the disease progresses, while detectability rates in general appear to decrease as the disease progresses and viral shedding occurs. These results suggest that NP and OP swabs are most reliable during early onset of the virus. In accordance with this, it has been reported that patients who have received negative results for both NP and OP swab tests have tested positive by other means such as bronchiolar lavage, as detailed in a research letter by Winichankoon, whereby a man in Thailand presented with the symptoms of COVID-19 and was repeatedly deemed negative *via* NP and OP swabs. After careful examination, a bronchoalveolar lavage was conducted and the man was found to be positive for COVID-19 [74]. Overall, it is evident from this study that NP tests are more reliable than OP tests but are subject to inaccuracy on both accounts in respect to detection and disease stage.

In another study carried out in Wuhan, 4480 suspected cases were tested using nucleic acid (Rt-PCR) detection methods. Of those 4480 cases, 1875 (38.42%) tested positive for SARS-CoV-2. Among these cases NP swabs exhibited a 39.80% positivity rate, while 40.98% of OP swabs were found to be positive [75]. While in contrast to the detectivity rates observed by Yang (2020), the range in observed differences is not as great. It is important to note that it is unclear what stage of the disease the patients were in as RT-qPCR is at its most sensitive within 7 days post-symptom onset [42]; and while the first study carried out by Yang (2020) shows high detectivity rates, the sample size used was smaller. A higher sample size allows for a more accurate observation into how well these methods work.

Upon review, it would suggest that general swabbing alone, by means of testing the NP and OP cavities, may prove to be inefficient in comparison to other methods, both individually and in tandem. It is important to reiterate that NP tests are still the recommended testing method by the CDC. Swabbing is considered invasive to the patient, it requires care and precision upon collection by the front-line staff and is open to shortages and PCR-interference issues based upon material. Swabbing also carries a level of risk to front-line staff of aerosolised transmission upon collection, and they are not easily self-collected by the patient. When compared and contrasted to other sampling methods, it can be said that having NP and OP swabs listed as a preferred collection method must be reassessed, along with the recommendation that multiple sample types should be collected if relying on the swabbing method. If patients are presenting with upper-respiratory symptoms such as a sore throat, runny nose or nasal congestion, then OTW, NP and OP swabs would be a suitable method of collection, as seen in Figure 2.

### 4.2. Bronchoalveolar Lavage Liquid Sample

Based on several studies, BLL is one of the most reliable samples to test for COVID-19, due to the nature of this disease. BLL samples were also collected during the same study conducted by Liu (2020) and Yang (2020). Firstly, within a hospital in Wuhan, China, only 5 patients within a cohort tested using BLL sampling methods (while not stated as to why, it can be speculated only 5 patients were intubated, making a BLL sample accessible) and an 80% positivity rate was observed [75]. Secondly, daily samples were taken from infected patients in Shenzhen Third Peoples hospital across 4 weeks. It was noted that BLL showed 100% positivity in all patients upon admission, followed by 95%, 82%, 72% and 54% detection over weeks 1, 2, 3 and 4, respectively [73].

Another set of samples was taken from 3 hospitals across the Hubei and Shandong provinces in China; 15 of these were BLL samples. It was observed how a 93% positivity rate was recorded in BLL samples in comparison to sputum, NP and OP samples (72%, 63% and 32%, respectively) [76].

**Figure 3:**
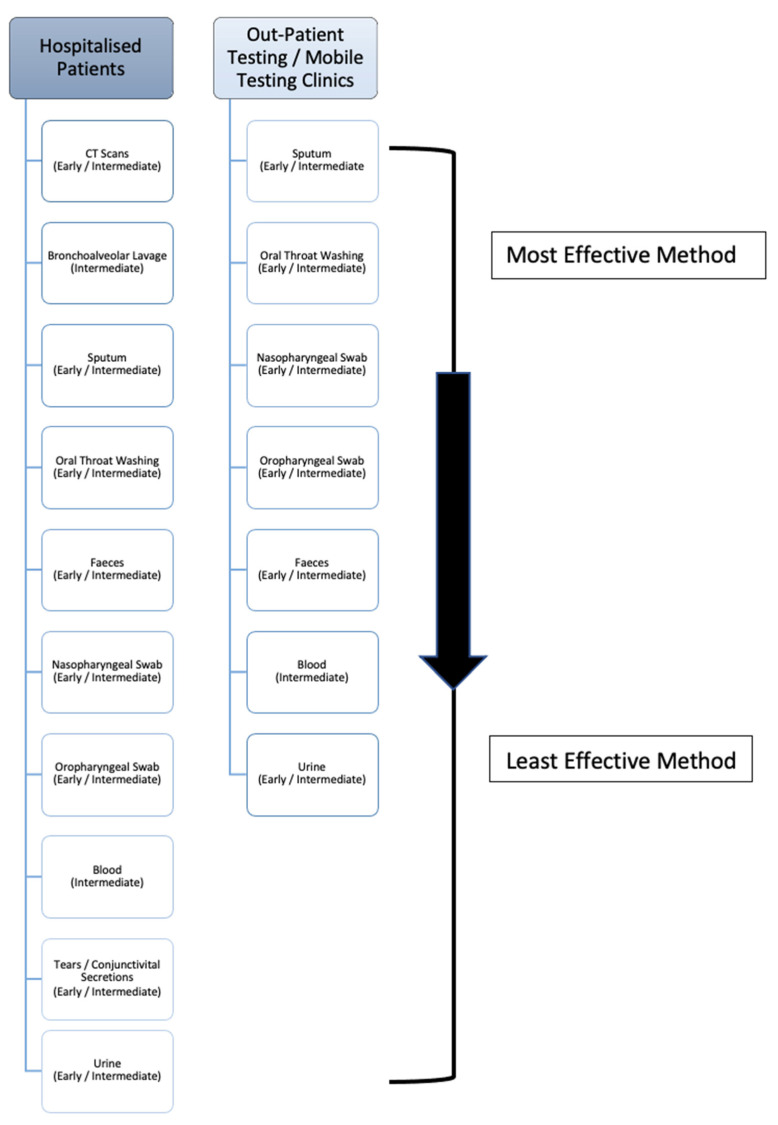
The deduced hierarchy in sample type and CT scan efficacy for diagnosis of COVID-19 disease, in both hospitalised patients and outpatient/mobile clinics along with the recommended disease-stage progression time-point to test at.

Upon review it must be noted, while the recorded percentage positivity rates of these BLL samples is high, the reported sample sizes are low in comparison to the other test methods due to the fact bronchoalveolar lavage testing is cumbersome and not routinely carried out unless a patient has typically been previously intubated. However, due to having direct access to the lungs, BLL has a high level of viral detection, and as seen in Figure 3 is the second best sampling method in hospitalised patients. Nonetheless, if patients are showing symptoms such as haemoptysis (a significant indicator of lung disease), as seen in Table 3, a bronchoalveolar lavage sample should be taken to both confirm the presence of COVID-19 and rule out the presence of any other disease/infection.

### 4.3. Sputum Samples

According to the WHO–China Join Mission Report on COVID-19, sputum production is observed in 33.7% of all patients who acquire the disease [78], suggesting that this sampling method has testing limitations. However, in patients that are actively producing the specimen, studies have shown that this sampling technique is successful in diagnosing patients [75]. In particular, 1875 patients from a hospital in Wuhan, China, were diagnosed with COVID-19. Different sampling methods were used and it was found that sputum samples exhibited a 49.12% positivity rate, in comparison to NP swabs which exhibited a 39.64% positivity rate [75]. Furthermore, a study by Yang et al. (2020) collected 866 samples from 213 infected patients in Shenzhen Third Peoples hospital. Samples were divided into groups based on stage of infection ranging from days 0 to 7, 8 to 14 and 15+ (Figure 2). Based on these results, sputum samples showed a high level of viral detection with a percentage positivity of 87.5%, while NP swabs showed a positivity rate of 72.7%. Even after 15+ days, sputum samples still exhibited a high level of positivity, ranging from 29.5 to 38.3% [73].

Upon review, despite only being produced in 33.7% (according to the WHO) of patients, sputum comes directly from the lungs, thus yielding a high level of detectivity. Based on the evidence provided, it can be said that sputum may be a more accurate specimen to collect in comparison to NP and OP swabs, when the patient is actively producing sputum. When compared to oral throat wash (OTW) samples, more studies must be carried out on OTW methods in order to accurately make a considerate statement when contrasting these upper-respiratory collection methods. As a result, sputum should be the primary sample collected in outpatient centres/mobile testing units, and is the third most effective sample for hospitalised patients (Figure 3), as it is relatively easy to collect and non-invasive. The nature of this method, unlike the previous, allows for self-collection which can help protect front-line workers and prevent viral transmission. Consequently, this is the most effective sample that outpatient and mobile clinic front-line workers with limited resources can collect. If patients are actively producing sputum via a productive cough, as reflected in Table 3, it could be recommended to sample the sputum instead of nasopharyngeal swabs or bronchoalveolar lavage liquid

### 4.4. Oral Throat Washing

While NP and OP swabbing is the preferred sampling method of the CDC and the World Health Organisation (WHO), it can be argued that OTW is another safer, less invasive and more efficient means of sample collection. Firstly, the production of live aerosolised virus during NP and OP sampling places front-line workers at risk; conversely, the self-collection of gargled solution prevents aerosol transmission. Secondly, the quality of swab sampling and stage-of-illness is open to significant variation, thus creating the possibility of false negatives; saline solution is standardised and throat washing covers a large surface area. Thirdly, swabbing is highly invasive and leads to patient discomfort; throat washing is a comfortable, non-invasive experience [79].

The OTW technique was carried out on 11 COVID-19-positive patients in China comprising 5 discharged patients and 6 hospitalised patients, with a median sample collection date of 53 days post-symptom onset (range: 48–57 days). A total of 24 paired (taken at the same time) OTW samples and NP samples were taken. While most of the paired samples came back as negative, 5 pairs had shown inconsistent results, whereby the OTW came back as positive, and the NP swabs came back as negative. Using statistical analysis, a statistically significant difference was observed between using OTW samples as opposed to NP swabs. While the sample size was small, the OTW sampling method was able to detect viral nucleic acid 48 days PSO [79].

The OTW method was observed to be a significant way of detecting SARS-CoV virus in 2004, with large recorded viral titre (9.58 × 10^2^ to 5.93 × 10^6^ copies/mL) yields. As a result, it was recommended by the study’s authors that OTW should be included in the collection guidelines for SARS diagnosis [80].

Upon review, the next most effective collection method is the oral throat washing sample. Again, this is non-invasive, allows for self-collection to prevent transmission and has a high level of detection due to the large surface area that the wash can cover within the oral cavity as it collects the sample. The oscillation of the cricopharyngeal muscle over posterior pharyngeal wall allows for the virus-containing mucus to be dislodged to an extent and efficiently gathered within the saline rinse. As a result, it has previously been recommended to be included in collection guidelines as outlined by Wang (2004). As seen in Figure 3, this is the second most effective sample collection method for mobile clinics and outpatient staff, and the fourth most effective sample collection method within hospitalised patients.

### 4.5. Faecal Sampling

For the purpose of uniformity, anal swabs will be included under the subsection of faecal sample (FS) collection. A study involved nine patients (noted as a limitation within the study) confirmed to have COVID-19, who were subjected to a range of sampling methods at different days PSO (post-symptoms onset) including anal swabs. Of the 9 patients, two showed viral titres from their anal swabs, both at day 3. Interestingly, not all patients that tested positive *via* anal swabs had diarrhoea, indicating that COVID-19 can invade the digestive system. Viral components that have been shed through the digestive tract have been detected in faeces and as a result may be transmitted via faecal-oral transmission, with evidence of viral nucleic acid being found within clinical stool samples [81,82].

Furthermore, a study by Wang, 2020, with 1014 patients, in Wuhan, had been confirmed to have the virus by Rt-PCR. A total of 153 of these patients had their faeces examined and 44 people (29%) had detectable levels of the virus within their stool samples [76].

Another study followed the detection of viral nucleic acid in patient stools upon admission and over the course of 4 weeks, across mild and severe symptom patients. Out of a total of 93 samples, upon admission, 55 (59%) tested positive for the virus. There was no observed difference between mild and severe cases in viral loads. Upon review of the cases, in both mild and severe cases, the amount of viral load present within the stool peaked upon admission, reduced over the first week, peaked again over weeks 2 and 3, and declined at week 4. While the administration of therapeutic care could affect these results, it appears that the shedding of viral load is high at the initial stages of infection, and again at weeks 2 and 3, which would indicate the best times for sample collection [83]. Similar sentiments were observed with the 2004 SARS virus, whereby a 13% detectivity of the virus was found upon admission, with peak levels of detectivity (70%) occurring at days 9–11, with a steady decline in the days thereafter [84].

Upon review, while the length and duration of the presence of the virus within the GI (gastro-intestinal) tract is unknown, some studies suggest it may be present longer than found within the respiratory system. Indeed, a study detected viral RNA in stool samples from paediatric patients for longer than 4 weeks [85]. While less sensitive than the previously listed sample types, evidence suggesting that viral RNA can be detected within faecal matter long after it has left the lungs is a highly relevant piece of information for two reasons. Firstly, it shows that the virus could still be transmitted via the faecal-oral route, even after the initial 2 week quarantine period, and secondly, it has a potential to be a highly useful diagnostic tool within a clinical setting during post-disease analysis of a patient. As a sampling method for detection, FS appears to be more conclusive than blood, urine and conjunctivital secretions for the detection of COVID-19, but less conclusive than some upper-respiratory samples. Upon examination of a total population of results, FS appear to be only slightly better than both NP and OP swabs in terms of the percentage of positive detection within the sample. Therefore, upon admission to hospital (as seen in Figure 3) patients should be sampled collectively *via* faecal sampling and both NP and OP swabs. For outpatient/mobile clinics, both NP and OP swabs are more efficient, followed by faecal samples. That being said, the persistent shedding of viral RNA allows for the possibility of longer term detection of the virus, should the necessity arise, which is not commonly seen in the other sample types and may be useful information within a clinical diagnostic setting. It should also be noted that carers of patients should be careful of possible faecal transmission of the virus on approximately days 9–14, where shedding is at its highest.

### 4.6. Other Potential Sample Types

In a study by Wang (2020), both urine and blood samples were taken from infected patients from 3 hospitals across the Hubei and Shandong provinces in China; 72 of which were urine samples (US), and 307 of which were blood samples (BS). Of the samples taken, none of the US came back as positive, and 3/307 (1%) of the BS came back positive [76].

Conversely, there is only one study carried out in which a patient tested positive via a US [81]. Nine patients, confirmed to have COVID-19, were subjected to a range of sampling methods at different days from the onset of their symptoms. These samples were subjected to Rt-PCR testing. Of the 9 patients, one showed viral titres (3.22 × 10^2^) in their urine at day 7. While the sample size is low, this may be due to a sampling error, but it must be noted that the effects of COVID-19 on the urinary system is not fully understood. In a second study by Peng et al. (2020), 2/9 patients showed viral titres within their blood samples. Interestingly, both patients’ samples were collected on day 3, yet the severity of their symptoms was not reported [81].

Upon review, blood, conjunctivital secretions and urine (in that order as seen in Figure 3), are the least effective samples to collect for diagnostic purposes within a hospital setting.

Though the research carried out is limited, from the given evidence, it appears that urine is the least useful sample (11% detection) from those examined to date while blood is also a highly unreliable source of testing for the virus (22% detection). As a result, blood and urine should not, in any setting, be used as a primary sampling method and should only be considered as an additional post hoc test having employed more effective methods as listed above.

According to the WHO–China joint mission on COVID-19 report, conjunctivital congestion is observed in 0.8% of all COVID-19 patients. While the lack of specimen production deems this method a highly unsuitable testing technique, it carries a level of caution as CS and tears can transmit the virus. At the First Affiliated Hospital of Zhejiang University, China, 30 COVID-19-confirmed patients had their tears and CS collected and subjected to RT-PCR analysis, over 2–3 day intervals. Of these patients, 21 had mild symptoms and 9 had severe symptoms. Only a single patient’s (1 out of 30) tears and CS tested positive for the virus while the rest tested negative. It was noted that all patients without CS tested negative [86]. A meta-analysis examining the levels of confirmed positivity via ocular swabs for the detection of COVID-19 found that the proportion of patients reporting conjunctivitis/red eye was 3.175% (95% CI 1.165–6.127). However, only 0.703% patients (95% CI 0.0358–3.269) reported conjunctivitis as the first symptom of the disease. Among all COVID-19 patients, the proportion of conjunctival/tear sample that was positive for the virus (RT-PCR detection of SARS-CoV-2) was found to be 1.949% (95% CI 0.743–4.113) [87]. Upon review, while the occurrence of this specimen is low and an unsuitable sampling technique, based upon statistical reliability when compared to the other sampling techniques, it carries a level of caution as a form of viral transmission.

## 5. Conclusions

Upon review, it is clear that many COVID-19-positive patients remain both undiagnosed and misdiagnosed despite undergoing the testing processes. Therefore, as discussed throughout this review, some key recommendations, capable of reducing the levels of undiagnosed and misdiagnosed patients and halting the spread of the virus, can be made under the following three key headings; sampling recommendations, commercial assay recommendations and transmission model recommendations.

Key sampling recommendations include that general swabbing of the NP and OP cavity alone is not enough to prevent false-negative results in those with low viral loads relating to disease stage or severity. Augmenting sample testing with a CT scan can greatly increase the likelihood of obtaining a positive diagnosis. Sputum, if produced, and oral throat washing samples give an assay more sensitivity than standard swab samples. The non-invasive nature of this collection method reduces the chances of community-based transmission to hospital workers in first-world and third-world countries where resources may be limited, and may even allow for an at-home self-collection service to become established. Health care staff dealing with faecal waste, especially care staff in nursing homes, should be made aware that the virus can remain in the GI tract longer than the upper-respiratory tract.

Commercial assay kit recommendations include the recognition that the novel Oxford antibody test should be used as the gold-standard antibody test due to its high sensitivity and specificity in comparison to the other commercial kits currently on the market. It was noted and recommended that these kits must only be used at specific timeframes post-symptom onset when antibody levels are at their highest to avoid false negatives.

This review also suggests a need for further research into the transmission from both symptomatic and asymptomatic patients while emphasising that children are unknowingly at risk of continuing the spread of the virus due having the highest levels of asymptomatic carriage.

## Figures and Tables

**Table 1:** Collation of numerous studies showcasing the number and percentage of patients presenting asymptomatic and symptomatic for diagnosis with SARS-CoV-2 infection.

No. of Patients and Case Studies	No. of Asymptomatic Patients (%)	No. of Symptomatic Patients (%)	Reference
628 patients 1 study	576 (92%)	52 (8%)	[6]
303 patients 1 study	110 (36.3%)	193 (63.7%)	[8]
50,155 patients 41 studies	7818 (15.6%)	42,297 (84.4%)	[7]
N/A patients 94 studies	* N/A (20%)	* N/A (80%)	[9]
213 patients 1 study	41 (19.2%)	172 (80.8%)	[10]

* Information not available.

**Table 2:** Different commercial antibody and antigen test kits used to detect COVID-19 followed by their reported sensitivity and specificity.

Test Used	Brand	Sensitivity (%)	Specificity (%)
Antibody Test			
SARS-CoV-2 IgG assay	Abbott	92.7	99.9
LIAISON SARS-CoV-2 S1/S2 IgG assay	DiaSorin	96.2	98.9
Elecsys Anti-SARS-CoV-2 assay	Roche	97.2	99.8
SARS-CoV-2 Total assay	Siemens	98.1	99.9
Novel 384-well ELISA	Oxford	99.1	99.0
Antigen Test			
*BinaxNOW™* COVID-19 rapid antigen test	Abbott	93.3	99.9
*SARS-CoV-2 Antigen Rapid Qualitative Test*	Innova	76.8	99.68
*LumiraDx* SARS-CoV-2 Antigen Test	Lumira	97.6	96.6

**Table 3:** The prevalence of COVID-19 within patients presenting with defined symptoms, contrasted against the percentage of those who experience these symptoms, all matched to the appropriate sample type to test for the disease [42,77].

Examination or Sample Type	Symptom(s) Associated with Choosing This Sample Type	Number of Studies	Number of People Sampled	% Prevalence of COVID-19 in Sampled Patients with Associated Symptoms	% Prevalence of Symptoms in the General Public Reporting to Be COVID-19 Positive
Chest-CT scan	Dry Cough	136	17,380	58	69.15
	Chest Pain	30	3510	7	N/A
Bronchoalveolar lavage	Haemoptysis	21	4698	2	0.48
Sputum	Productive Cough	136	17,380	58	27.88
Faeces	Diarrhoea	93	11,707	10	9.96
Oral throat wash, nasopharyngeal swab and oropharyngeal swab	Sore Throat	78	11,721	12	9.55
	Runny Nose	36	10,656	8	N/A
	Nasal Congestion	10	2584	5	3.36
Conjunctivital secretions	Conjunctivitis	9	2715	2	0.55

## Data Availability

All data referenced in this review are cited in this published article.

## Abbreviations

AI	Artificial Intelligence
BLAST	Basic Local Alignment Search Tool
BLL	Bronchoalveolar Lavage Liquid
BS	Blood Sample
CDC	Centre for Disease Control (America)
COVID-19	Coronavirus Disease-2019
CS	Conjunctivital Secretions
CT	Computed Tomography
Ct	Cycle Threshold
ELISA	Enzyme-Linked Immunosorbent Assay
FS	Faecal Sample
GI	Gastrointestinal
IgM	Immunoglobulin-M
LDTs	Laboratory-Developed Tests
NP	Nasopharyngeal
OP	Oropharyngeal
OTW	Oral-Throat Wash
PCR	Polymerase Chain Reaction
PSO	Post-Symptoms Onset
RdRp	RNA-Dependent RNA Polymerase-Encoding
RNA	Ribonucleic Acid
RT-qPCR	Real-Time Polymerase Chain Reaction
SARS-CoV-2	Severe Acute Respiratory Syndrome Coronavirus 2
SARS	Severe Acute Respiratory Syndrome
US	Urine Sample
VTM	Viral Transport Media
WHO	World Health Organisation
